# White Matter Microstructure Alterations in Patients With Spinal Cord Injury Assessed by Diffusion Tensor Imaging

**DOI:** 10.3389/fnhum.2019.00011

**Published:** 2019-02-12

**Authors:** Yun Guo, Feng Gao, Yaou Liu, Hua Guo, Weiyong Yu, Zhenbo Chen, Mingliang Yang, Liangjie Du, Degang Yang, Jianjun Li

**Affiliations:** ^1^School of Rehabilitation Medicine, Capital Medical University, Beijing, China; ^2^China Rehabilitation Research Center, Department of Spinal and Neural Functional Reconstruction, Beijing, China; ^3^Center of Neural Injury and Repair, Beijing Institute for Brain Disorders, Beijing, China; ^4^China Rehabilitation Science Institute, Beijing, China; ^5^Beijing Key Laboratory of Neural Injury and Rehabilitation, Beijing, China; ^6^Department of Radiology, Beijing Tiantan Hospital, Capital Medical University, Beijing, China; ^7^Center for Biomedical Imaging Research, Department of Biomedical Engineering, School of Medicine, Tsinghua University, Beijing, China; ^8^China Rehabilitation Research Center, Department of Radiology, Beijing, China

**Keywords:** spinal cord injury, cerebral white matter microstructure, diffusion tensor imaging, tract-based spatial statistics, atlas-based analysis

## Abstract

Compared to healthy controls, spinal cord injury (SCI) patients demonstrate white matter (WM) abnormalities in the brain. However, little progress has been made in comparing cerebral WM differences between SCI-subgroups. The purpose of this study was to investigate WM microstructure differences between paraplegia and quadriplegia using tract-based spatial statistics (TBSS) and atlas-based analysis methods. Twenty-two SCI patients (11 cervical SCI and 11 thoracic SCI) and 22 age- and sex-matched healthy controls were included in this study. TBSS and atlas-based analyses were performed between SCI and control groups and between SCI-subgroups using multiple diffusion metrics, including fractional anisotropy (FA), mean diffusivity (MD), axial diffusivity (AD) and radial diffusivity (RD). Compared to controls, SCI patients had decreased FA and increased MD and RD in the corpus callosum (CC; genu and splenium), superior longitudinal fasciculus (SLF), corona radiata (CR), posterior thalamic radiation (PTR), right cingulum (cingulate gyrus; CCG) and right superior fronto-occipital fasciculus (SFOF). Cervical SCI patients had lower FA and higher RD in the left PTR than thoracic SCI patients. Time since injury had a negative correlation with FA within the right SFOF (*r* = −0.452, *p* = 0.046) and a positive association between the FA of left PTR and the American Spinal Injury Association (ASIA) sensory score (*r* = 0.428, *p* = 0.047). In conclusion, our study suggests that multiple cerebral WM tracts are damaged in SCI patients, and WM disruption in cervical SCI is worse than thoracic injury level, especially in the PTR region.

## Introduction

Spinal cord injury (SCI) is a devastating neurological disease characterized by motor and sensory deficit below the injury level (Dietz and Curt, 2006; Min et al., 2015). Patients with SCI usually suffer from paraplegia or quadriplegia due to the disruption of efferent motor and afferent sensory pathways between the brain and periphery (Jurkiewicz et al., 2010). Although researchers have tried for decades to explore the mechanism of neural repair and functional reorganization in basic research and clinical trials, an effective and reliable clinical strategy for SCI patients has yet to be determined (Dietz and Curt, 2006). As a traumatic injury to the central nervous system, SCI inevitably involves damage to white matter (WM) and causes primary mechanical destruction of glia and axons (Matute and Ransom, 2012). Wallerian degeneration of WM tracts occurs and spreads in both anterograde and retrograde directions from the injury site in spinal pathways after injury (Buss et al., 2004), and the progressive and enduring neurodegenerative and plastic processes induced by SCI even at the supraspinal level (Guleria et al., 2008; Ziegler et al., 2018).

Recent advances in imaging and analysis techniques have made subtle changes in brain WM after SCI potentially measurable. For example, diffusion tensor imaging (DTI) allows for measuring the diffusion of water molecules in tissues and provides quantitative information on tissue microstructures (Bodini et al., 2009). In particular, fractional anisotropy (FA) is related to fiber loss, while mean diffusivity (MD) measures the diffusion rate of molecules in selected voxels (Solstrand Dahlberg et al., 2018). Both of these techniques are believed to reflect WM integrity and underlying pathologies (Song et al., 2003; Ramu et al., 2008; Nagahara et al., 2014) and are commonly combined employed in studies of brain diseases (Wei et al., 2008; Ferris et al., 2017).

DTI imaging studies observed changes in WM microstructure in the brain in SCI patients compared to healthy controls even with heterogeneous results. Wrigley et al. (2009) showed reduced FA and increased MD values in SCI subjects with injury to the thoracic level compared to controls. In particular, the corticospinal and corticopontine tracts showed reduced FA values in SCI subjects. Freund et al. (2012) also reported decreased FA values in the cranial corticospinal tract in patients with a cervical SCI. Zheng et al. (2017) suggested that lower FA was found in several cerebral WM tracts in SCI patients, such as left angular gyrus, right cerebellar, left precentral and postcentral WM, left superior longitudinal fasciculus (SLF). Some studies have reported widespread changes in MD values in the brain in SCI subjects with and without neuropathic pain (Gustin et al., 2010; Yoon et al., 2013). However, Wei et al. (2008) and Henderson et al. (2011) presented no significant regional FA differences between the SCI group and healthy controls.

Most studies generally compared the SCI patients with healthy controls and have demonstrated reduced WM integrity in SCI patients, but little progress has been made in investigating whether cerebral WM differences happen between SCI-subgroups, such as paraplegia and quadriplegia or between patients with complete and incomplete SCI. Functional preservation in SCI individuals depends on their injury levels and severities. Functional brain neuroimaging studies have provided evidence that cortical reorganization or brain activation in the sensorimotor cortex in SCI populations is based on functional preservation. Sabre et al. (2016) reported that during hand movements, the volume of activation (VOA) in the contralateral primary motor cortex was significantly larger among the thoraco-lumbar SCI patients, but that VOA did not enlarge during ankle movements. They considered that cortical activation in the chronic phase of thoraco-lumbar SCI may be caused by increased use of upper limbs. Similarly, it is possible for cerebral WM microstructures to change in SCI patients with different functional preservations based on these functional studies. It would be helpful to elucidate the mechanisms of neurodegeneration in cerebral WM in paraplegia and quadriplegia following SCI for the development of future treatments and rehabilitation. Given that there is no study directly comparing WM microstructure changes in the brain across SCI-subgroups, we proposed an imaging study to investigate WM microstructural differences between paraplegia and quadriplegia using tract-based spatial statistics (TBSS) and atlas-based analysis methods. We hypothesized: (1) that WM microstructural abnormalities occur in SCI patients and differences in these exist in patients with cervical and thoracic injuries; and (2) that these changes may be related to post-SCI neurodegeneration or existing brain function.

## Materials and Methods

### Subjects

Twenty-two SCI patients (11 with cervical injury, 11 with thoracic injury) and 22 healthy controls; all right-hand dominant, participated in the current study. All SCI patients were recruited from China Rehabilitation Research Center. The extent of motor and sensory impairment was assessed by a qualified clinician (Dr. Gao) using the American Spinal Injury Association (ASIA) classification scale. No patients suffered from a psychiatric disorder. All the SCI patients were diagnosed as without concomitant brain injury based on MRI scans of the brain. Healthy controls were recruited by advertisements on the Internet. None of the controls had neurological or psychiatric diseases. All the subjects had no contraindications to MRI. The SCI patients and healthy controls were matched for age and gender. Clinical and demographic data from all participants are shown in Table 1.

**Table 1:** Demographic and clinical characteristics of SCI patients and healthy controls.

Characteristics	SCI (*n* = 22)	Controls (*n* = 22)	CSCI (*n* = 11)	TSCI (*n* = 11)	*P* value SCI vs. Cont	*P* value CSCI vs. TSCI
Handedness (right:left)	22:0	22:0	11:0	11:0	1	1
Gender (male:female)	19:3	18:4	10:1	9:2	0.68	0.53
Age (years, mean ± SD)	40.23 ± 10.04	41.73 ± 6.58	40.18 ± 11.11	40.27 ± 9.39	0.56	0.98
ASIA level (cervical:thoracic)	11:11	-	11:0	0:11		
Etiology (No.)						
Vehicle accident	5	-	2	3	-	-
Fall	5		3	2		
Crush by weight	9		5	4		
Sport injury	2		1	1		
Others	1		0	1		
Months since injury (mean ± SD)	45.32 ± 44.47	-	44.82 ± 44.01	45.82 ± 47.07	-	0.96
ASIA total motor score (mean ± SD)	42.05 ± 22.17	100 ± 0	29.82 ± 23.16	54.27 ± 12.90	<0.001	0.006
ASIA total sensory score (mean ± SD)	101.86 ± 51.73	224 ± 0	63.91 ± 44.84	139.82 ± 20.96	<0.001	<0.001
SCIM score (mean ± SD)	42.18 ± 17.49	100 ± 0	29.27 ± 13.34	55.09 ± 9.88	<0.001	<0.001

*SCI, Spinal Cord Injury; Cont, Control; CSCI, Cervical SCI; TSCI, Thoracic SCI; ASIA, American Spinal Injury Association; SCIM, Spinal Cord Independence Measure*.

The study was carried out in accordance with the recommendations of “the medical ethics committee of China Rehabilitation Research Center” with written informed consent from all subjects. All subjects gave written informed consent in accordance with the Declaration of Helsinki. The protocol was approved by the “the medical ethics committee of China Rehabilitation Research Center”.

### Magnetic Resonance Imaging Data Acquisition

All subjects were scanned on a 3T MRI scanner (Philips Ingenia, Best, Netherlands) at the China Rehabilitation Research Center, Beijing, China. We used a single shot echo-planar imaging (EPI) sequence in contiguous axial planes covering the whole brain for the DTI. Scanning parameters were: repetition time (TR) = 5,924 ms, echo time (TE) = 102 ms, flip angle = 90°, one image with *b* = 0 s/mm^2^ and 32 non-collinear directions with *b* = 2,000 s/mm^2^, thickness slices = 2.8 mm, slice gap = 0, field of view _(FOV)_ = 224 × 224 mm, matrix size = 80 × 78.

### Image Preprocessing

All DTI image preprocessing and analyses were executed using a pipeline tool for diffusion MRI (PANDA; Cui et al., 2013). The main procedure was conducted as follows: first, DICOM files for all subjects were converted into NIfTI format using the dcm2nii tool embedded in MRIcron. Second, the brain mask was yielded by estimating the b0 image without diffusion weighting. Third, the non-brain space in the raw images was separated to reduce the memory cost, speed up the processing in subsequent steps, and generate a reduced image size. Fourth, the diffusion-weighted image (DWI) was registered to the b0 image with an affine transformation to correct eddy-current induced distortions and simple head motion artifacts. The gradient direction of each DWI volume was rotated according to the resultant affine transformations. Fifth, a voxel-wise calculation of the tensor matrix and the diffusion tensor metrics were yielded, including FA, MD, axial diffusivity (AD), and radial diffusivity (RD).

### Tract-Based Spatial Statistics (TBSS)

The TBSS analyses of FA, MD, AD, and RD images were carried out using the FMRIB software library (FSL 5.0.0^1^). Briefly, for the FA images, the following five-step process was performed: (1) the FA image of each subject was aligned to a pre-identified target FA image (FMRIB58_FA) by nonlinear registration; (2) all the aligned FA images were transformed to the 1 × 1 × 1 mm Montreal Neurological Institute (MNI152) standard space using affine registration; (3) the mean for all aligned FA images was created and skeletonized, resulting in a mean FA skeleton; (4) all individual subjects’ aligned FA maps were projected onto the mean FA skeleton; and (5) voxel-wise statistical analysis across subjects was calculated for each point on the skeleton. Corresponding skeletonized maps and voxel-wise cross-subject statistics for MD, AD and RD were similarly performed.

In this study, voxel-wise inter-group comparisons (Control vs. SCI) and intra-group comparisons (Cervical SCI vs. Thoracic SCI) were tested in the general linear model (GLM) framework with an unpaired *t*-test, using the FSL randomize tool with nonparametric permutation testing, controlling for age and gender during inter-group comparison and age, gender, and months since injury during intra-group comparison. The mean FA skeleton was used as a mask (thresholded at a mean FA value of 0.2), and the number of permutations was set to 5,000. The significance threshold for between-group differences was set at *P* < 0.05 [family-wise error (FWE) corrected for multiple comparisons] using the threshold-free cluster enhancement (TFCE) option in the “randomize” permutation-testing tool in FSL. Similar comparisons of MD, AD and RD images were performed.

### Atlas-Based Analysis at the Tract Level

**Figure 1:**
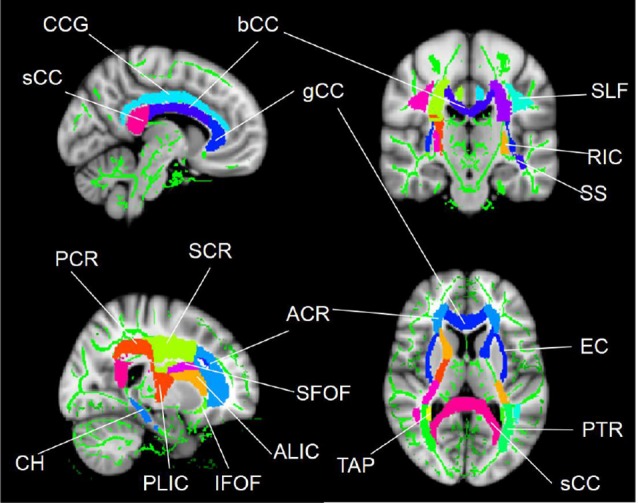
WM tract regions of interest (ROIs) based on the ICBM-DTI-81 WM labels atlas in cerebral regions. Abbreviations for WM tracts can be seen in Supplementary Table S1.

The regions that exhibited alterations in the FA due to SCI were defined as regions of interests (ROIs; Figure 1) using the ICBM-DTI-81 WM labels atlas^2^, which is a probabilistic atlas generated by mapping DTI data of 81 subject to a template image. The mean diffusion metric values of each ROI for each subject were extracted. Abbreviations for ROIs can be seen in Supplementary Table S1.

### Statistical Analysis

Statistical analysis was performed using SPSS software, version 20.0. Continuous variables were tested using two-tailed *t*-tests, while gender differences were examined by chi-square tests. *P* < 0.05 were considered to be statistically significant. We compared FA, MD, AD and RD values between subjects groups (Control vs. SCI) and within-subjects groups (Cervical SCI vs. Thoracic SCI) using a univariate ANCOVA model with FDR correction for multiple comparisons, controlling for age and gender between group comparisons and controlling for age, gender and time since injury in SCI-subgroup comparisons. Partial correlation analyses between DTI metrics and clinical data were performed to investigate the relationships between DTI-derived indices and clinical variables in SCI patients, using a significance level of 0.05.

## Results

### Demographics

Demographic and clinical data of SCI patients and healthy controls are shown in Table 1. There were 22 SCI patients (11 with cervical injury and 11 with thoracic injury) and 22 healthy controls in the study. All the subjects were right-handed. No statistically significant differences were found between SCI patients and healthy controls or within SCI-subgroups in age and gender (*p* > 0.05). The mean period post-SCI was 45.32 ± 44.47 months (range 1–139 months). The mean period of cervical SCI was 44.82 ± 44.01 months (range 2–133 months) and thoracic SCI was 45.82 ± 47.07 months (range 1–139 months). For the SCI patients, ASIA total motor score, ASIA total sensory score and spinal cord independence measure (SCIM) scores were 42.05 ± 22.17, 101.86 ± 51.73 and 42.18 ± 17.49, respectively. Cervical SCI patients had significantly lower motor, sensory and SCIM scores than the thoracic SCI patients (*p* < 0.05).

### TBSS Analysis

#### Group Differences Between SCI and Controls

**Figure 2:**
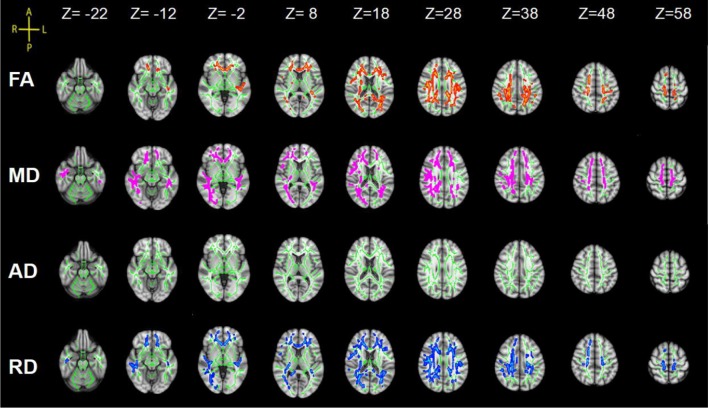
Tract-based spatial statistics (TBSS) results of fractional anisotropy (FA), mean diffusivity (MD), axial diffusivity (AD) and radial diffusivity (RD) images between the spinal cord injury (SCI) group and control (Cont) group. The background is based on MNI152_T1_1 mm. Green represents the mean white matter (WM) skeleton of all subjects; red color, pink and blue (the skeletonized results are “thickened” for easier visualization) represent regions with decreased FA, increased MD and increased RD in SCI patients respectively [*p* < 0.05, family-wise error (FWE) corrected for multiple comparisons].

Statistical analysis revealed significant group differences in diffusion metrics for the WM skeletons. Compared to controls, lower FA in SCI was found in the corpus callosum (CC; genu, body and splenium), bilateral SLF, bilateral anterior corona radiata (ACR), bilateral posterior corona radiata (PCR), bilateral superior corona radiata (SCR), bilateral posterior thalamic radiations (PTRs), internal capsule (IC; except the left posterior limb of IC), external capsule (EC), cingulum (cingulate gyrus; CCG), left sagittal stratum (SS), left tapetum (TAP), left cingulum (hippocampus; CH), right superior fronto-occipital fasciculus (SFOF), and right inferior fronto-occipital fasciculus (IFOF). Meanwhile, WM tracts of increased MD and RD were similar to FA. In contrast, no significant differences in AD were found between these two groups (Figure 2, Table 2).

**Table 2:** Diffusion metrics changes in the WM tracts in SCI patients using the TBSS and atlas-based statistical analysis.

WM tracts	TBSS statistical analysis (*P* < 0.05, FWE)			Atlas-based statistical analysis (*P* < 0.05, FDR)	
	FA	MD	RD	AD	FA
CC	g, b, s	g, b, s	g, b, s	-	**g, s**
SLF	bilateral	bilateral	bilateral	-	**bilateral**
ACR	bilateral	bilateral	bilateral	-	**bilateral**
SCR	bilateral	bilateral	bilateral	-	**R**
PCR	bilateral	bilateral	bilateral	-	**bilateral**
PTR	bilateral	bilateral	bilateral	-	-
RIC	bilateral	bilateral	bilateral	-	-
SS	L	bilateral	bilateral	-	-
CCG	bilateral	R	R	-	**R**
PLIC	R	R	R	-	-
EC	bilateral	bilateral	R	-	-
ALIC	bilateral	bilateral	-	-	-
TAP	L	bilateral	bilateral	-	-
SFOF	**R**	-	-	-	bilateral
CH	L	-	-	-	-
IFOF	R	R	bilateral	-	-

*WM, white matter; g, b, s: genu, body and splenium of the CC; L, left; R, right. Bold font represents common WM tracts in both TBSS and Atlas-based analysis. Abbreviations of WM tracts can be seen in Supplementary Table S1*.

#### Subgroup Differences Between Cervical SCI and Thoracic SCI Patients

TBSS analysis showed significant differences in bilateral PTR with decreased FA and increased RD in the SCI-subgroup [cervical SCI (CSCI) patients vs. thoracic SCI (TSCI) patients] (Figure 5). However, no differences were found in MD and AD.

**Figure 3:**
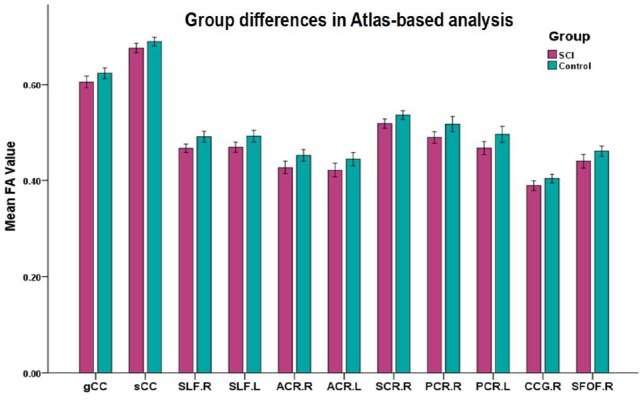
Mean diffusion metrics of the atlas-based tracts in SCI and healthy control groups. All the WM tracts shown were significantly different after FDR correction. Abbreviations of WM tracts can be seen in Supplementary Table S1.

**Figure 4:**
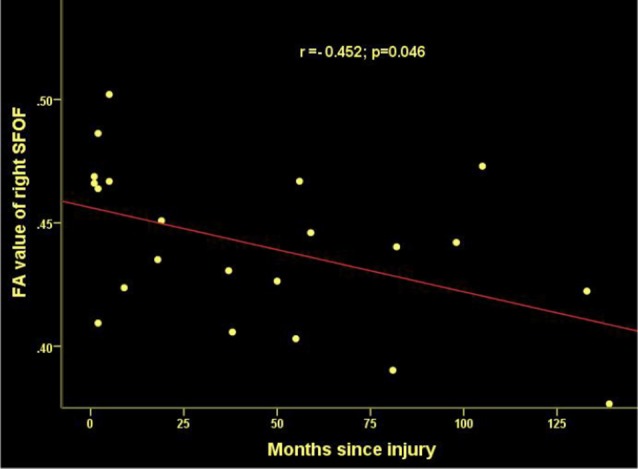
Significant correlations between months since injury and FA values in the right superior frontooccipital fasciculus (SFOF) in patients with SCI.

**Figure 5:**
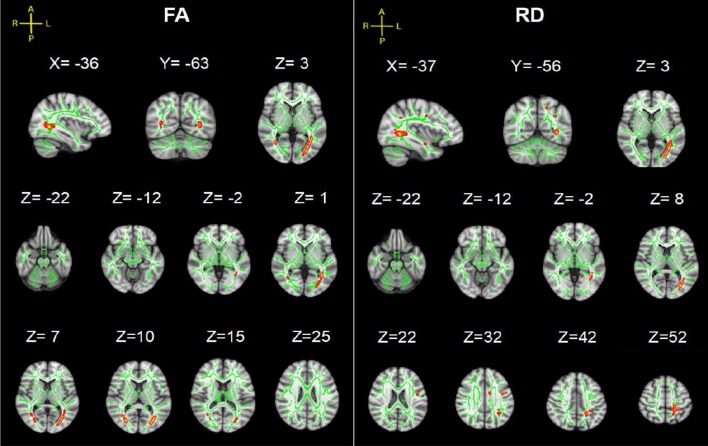
TBSS results of FA and RD images between cervical SCI (CSCI) and thoracic SCI (TSCI) groups. The background is based on MNI152_T1_1 mm. Green represents the mean WM skeleton of all SCI patients; red-yellow (the skeletonized results are “thickened” for easier visualization) represents regions with decreased FA and increased RD in CSCI patients (*p* < 0.05, FWE corrected for multiple comparisons).

### Atlas-Based Analysis

#### Group Differences Between SCI and Controls

Significant differences in lower FA were found in 12 ROIs in patients compared to controls (after FDR correction; Figure 3), including the CC (genu and splenium), bilateral SLF, bilateral ACR, bilateral SCR, right PCR, bilateral PTR, right CCG and bilateral SFOF (Table 2).

#### Subgroup Differences Between Cervical SCI and Thoracic SCI Patients

**Figure 6:**
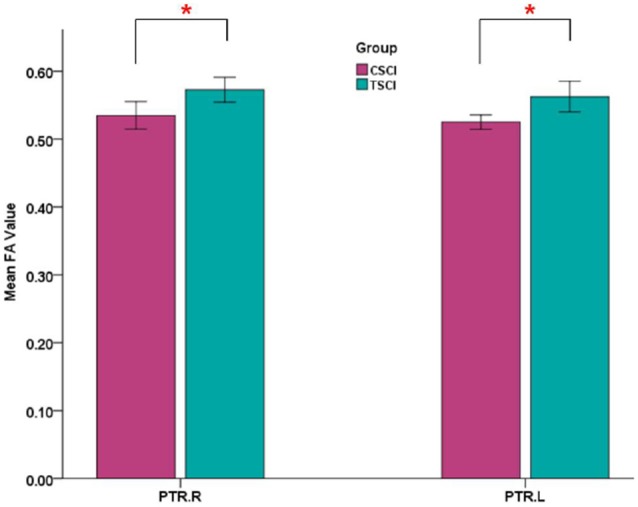
Mean FA values of bilateral posterior thalamic radiations (PTRs) in CSCI and TSCI. CSCI, cervical spinal cord injury; TSCI, thoracic spinal cord injury; *P < 0.05.

Significant differences in bilateral PTR with decreased FA and increased RD in the SCI-subgroup (cervical SCI (CSCI) patients vs. thoracic SCI (TSCI) patients; after FDR correction; Figure 6). However, no differences were found in MD and AD.

### Correlations Analysis

**Figure 7:**
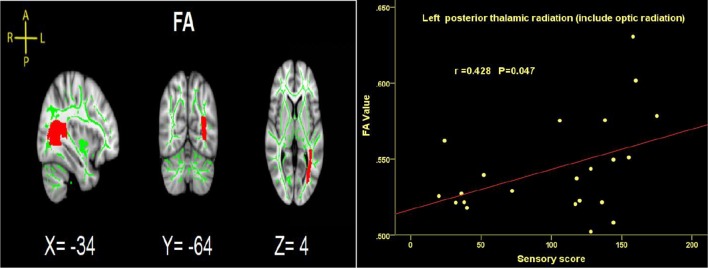
Significant correlations between total sensory score and FA values in the left PTRs in SCI patients.

Time since injury had a negative correlation with FA values within right SFOF using partial correlation analysis, controlling for age, gender and nuisance covariates (*r* = −0.452, *p* = 0.046. Figure 4). Pearson correlation showed a positive association between the FA of left PTR and the ASIA sensory score (*r* = 0.428, *p* = 0.047, Figure 7).

## Discussion

The present study investigated the changes of cerebral WM microstructures in patients with SCI and differences between CSCI and TSCI using TBSS and atlas-based analysis. Our results demonstrated that diffusion metrics of brain WM are altered in SCI patients and significant differences in cerebral WM were found between paraplegia and quadriplegia patients. Compared to healthy controls, FA decreased in SCI patients, while MD and RD increased in several brain WM tracts. When the SCI-subgroup comparison was performed between cervical and thoracic injury patients, higher FA and lower RD values were observed in the left PTR in thoracic injury patients. Meanwhile, we found a negative correlation between time since injury and FA of right SFOF, and the ASIA sensory score positively correlated with FA of the left PTR in SCI patients.

### Group Differences Between SCI and Controls

Afferent sensory and efferent motor pathways are disrupted after SCI. Multilevel changes have been shown not only at the site of injury, but functional and structural alterations of the distal brain are also seen in patients with SCI (Min et al., 2015). In previous DTI studies, various cerebral WM microstructure impairments were found in SCI patients when compared to healthy controls. Yoon et al. (2013) suggested that WM changes may be associated with abnormal sensory perception and motor impairment. However, it is unclear whether or how brain WM reacts to different extents of remaining sensory and motor functions after SCI. In order to investigate changes in cerebral WM microstructures in SCI patients with different residual function, we compared cerebral WM between SCI and controls and between cervical and thoracic SCI in our study mainly by TBSS analysis. TBSS, which uses non-linear image transformation, is a newly developed technique that combines the strength of both voxelwise and tractography-based analyses (Smith et al., 2006). It has been used to assess changes in cerebral WM integrity associated with various pathological processes in several diseases, including multiple sclerosis (Liu et al., 2012), Parkinson’s disease (Li et al., 2018), Alzheimer’s disease (Mayo et al., 2017) and schizophrenia (Nazeri et al., 2013). FA and MD are crucial diffusion metrics of WM microstructures in TBSS analysis.

Our TBSS analysis showed that SCI patients had lower FA in the CC, SLF, CR, PTR, IC (except the left PLIC), EC, CCG, SS (left), TAP (left), CH (left), SFOF (right), IFOF (right). WM tracts of increased MD and RD were similar to FA. These diffusion metrics changes indicated that WM pathological changes occurred in the brain after SCI. To date, there are several studies suggesting WM microstructure impairments in SCI patients. Zheng et al. (2017) reported lower FA in the distributed brain WM in SCI patients compared to controls, including in the left angular gyrus, right cerebellum, left precentral gyrus, left lateral occipital region, left SLF, left supramarginal gyrus, and left postcentral gyrus. Freund et al. (2012) presented decreased FA values in the corticospinal tract in the brain in individuals with cervical SCI. Wrigley et al. (2009) demonstrated SCI subjects had reduced FA in the corticospinal and the corticopontine tracts. In addition, we found MD and RD values increased significantly in SCI patients, but did not show between-group differences in AD. Therefore, the changes in FA and MD may be mainly driven by the increase in RD but not AD. Based on basic research (Song et al., 2003), increased RD is a marker for destruction of myelin integrity while AD is more related to axonal injury, our results imply that demyelination caused by degeneration may be an important contributing factor to WM alterations in SCI. Our study also found a negative correlation between disease duration and FA values in SFOF, suggesting a possible evolution of pathology in WM after injury.

Some degenerative diseases primarily affecting the spinal cord as hereditary spastic paraplegia and Friedreich ataxia (FRDA) have also shown WM microstructure changes in the brain. Dreha-Kulaczewski et al. (2006) reported reduced FA and increased MD in frontal and occipital inhereditary spastic paraplegia compared to controls. FRDA patients had significantly higher MD than controls in the medulla, pons, middle and superior cerebellar peduncle, pyramidal tract at the posterior limb of IC (PLIC) level, and optic radiation (Rizzo et al., 2011). The greatest decreases in FA were in the left superior cerebellar peduncle, left PTRs, major forceps, left IFOF and CC in FRDA patients (Vieira Karuta et al., 2015). These findings indicate damage to myelinated axons as both axonal membranes and myelin sheaths (Dreha-Kulaczewski et al., 2006). The similar brain WM changes of decreased FA and increased MD in degenerative diseases suggest physiopathological similarities to the group with injured spinal cords. Therefore, a comparison of brain WM changes between diseases in injured and degenerated spinal cords might provide more information in the future.

Meanwhile, we calculated the mean value of DTI metrics of ROIs by atlas-based statistical analysis. Significant differences in lower FA were found in 12 ROIs in patients compared to controls (after FDR correction). Combing both TBSS and atlas-based analyses, SCI patients presented WM alterations in CC (genu and splenium), bilateral SLF, bilateral ACR, bilateral SCR, right PCR, bilateral PTR, right CCG and right SFOF.

As the largest commissural WM bundle in the brain, the CC is topographically organized with its genu connecting orbitofrontal and frontal cortices, while its body and splenium connect temporal, parietal and occipital regions (Abe et al., 2004). The CC not only provides an interhemispheric connection to contralateral homologous brain systems, but fibers in the splenium of the CC also connect regions of the parietal cortex involved in somatosensory information processing (Caminiti et al., 2013). This study indicated that WM microstructural anomalies in the CC after SCI, which might be related to loss of sensory information input or imbalances in interhemispheric communication. SCI patients showed WM microstructure alterations in SLF, which is in accord with the previous study (Yoon et al., 2013; Zheng et al., 2017). The SLF is the longest fiber tract among the association of fiber bundles, and connects the frontal parietal, occipital and temporal lobes in the brain. The SLF is involved in regulating motor behavior, transferring somatosensory information, and contributing to the cortico-cerebellar system (Wang et al., 2016). DTI-derived metric changes in the SLF imply abnormal connectivity or that the brain network might be damaged or weakened across brain regions, which needs further investigation on functional connectivity to confirm. The ACR, SCR and PCR form part of the CR which is an important region passing through motor fibers arising from the human motor cortex to the posterior limb of the IC. Studies showed that CR infarcts can result in executive dysfunction (Han et al., 2010). Decreased FA in bilateral ACR, bilateral SCR and right PCR might be associated with motor impairments in SCI patients. PTR connects the thalamus with the posterior visual brain. The FA changes in PTR suggest the possibility of abnormalities in thalamus-visual connections in patients with SCI. WM changes in the PTR after SCI may be caused by the loss of the appropriate afferent feedback. The CCG is involved in working memory. Several studies report working memory decreases in SCI patients (Jensen et al., 2007). Therefore, impairment to working memory in SCI may be linked to CCG deficits.

A complex cascade of secondary neurodegenerative processes occurs across the spinal cord and brain following SCI (Huber et al., 2015). The regions mentioned above involved sensory or motor processing directly or indirectly. The post-SCI state may therefore be partially responsible for the diffusion metric changes in these brain regions. We thus speculate that the observed changes in the microstructures of WM might be associated with loss of motor output and sensory input, as well as demyelination due to SCI.

### Subgroup Differences Between Cervical SCI and Thoracic SCI Patients

More importantly, our study is the first to investigate brain WM microstructural differences between cervical and thoracic injury levels. The TBSS findings in the current study were that cervical SCI patients had lower FA and higher RD in the PTR compared with thoracic injury patients. This finding indicated that WM integrity of PTR in cervical injury is worse than that in thoracic injury. The PTR connects the thalamus and parietal/occipital lobes and is mostly related to sensory function (Nagae et al., 2007). Sensory information from the periphery is initially processed in the thalamus, with diffuse projections to the cortex, including the parietal-occipital cortex, by PTR (Hoon et al., 2009). The lower FA of PTR in cervical SCI suggests worse abnormalities in thalamus-visual connections than thoracic SCI. The differences in WM changes in the PTR between cervical and thoracic SCI may be caused by worse appropriate afferent feedback in cervical SCI. In order to investigate the relationship between the PTR and clinical parameters, we performed correlation analysis between FA in PTR and sensory, motor and SCIM scores, and found that the sensory score had a positive correlation with FA values in the left PTR. That is, the higher sensory score, the better WM integrity in SCI patients. Freund et al. (2012) also reported that the FA reduction in the corticospinal tract in the brain correlated with upper limb ability in patients with cervical SCI. Furthermore, based on the comparison between SCI and controls, abnormal cerebral WM occurrence in SCI patients may be associated with demyelination, deficits of sensory and motor function, and disease duration. Differences in cerebral WM between cervical and thoracic injuries may be driven by distance from the injury site to the brain, residual function below the injury level, and time since injury. Animal research suggests that the DTI parameters are affected by distance from the injury site (Liu et al., 2018). As cervical injury sites are closer to the brain, the brain WM demyelination in this type of patients might be lesser than in thoracic injury, because thoracic patients might need longer to adapt, but this needs more basic research to confirm. Additionally, residual function below the injury level may be another factor in this difference. In our study, the sensory, motor and SCIM scores in patients with cervical injury were significantly lower than in thoracic injury patients, which may play a role in worse WM microstructural integrity seen in cervical injuries. Finally, changes in cerebral WM as a result of spinal afferent denervation may develop over a period of time, and cerebral WM may be different at different injury times. However, time since injury in our SCI-subgroup comparison between the two injury levels had no significant difference.

Our study still has some limitations. The SCI patients in the current study had a broad range of disease durations, but patients in the SCI-subgroup comparison between cervical and thoracic injury were relatively homogeneous in time since injury. Moreover, due to the relatively small sample size in the SCI-subgroup, future studies may reexamine this subgroup with a larger cohort. In addition, due to variances in the level of injury, severity of injury, and ASIA classifications, we performed the SCI-subgroup comparison only on cervical and thoracic injury levels, so future studies should perform more SCI-subgroup comparisons. Finally, the exact histopathological processes leading to changes in DTI metrics over time are complex, and appropriate animal research and longitudinal studies will be useful to elucidate this in the future.

## Conclusion

In conclusion, our study provided imaging evidence for cerebral WM microstructural abnormalities in SCI patients, which are likely caused by demyelination and may be associated with abnormal sensory perception and motor impairment. Furthermore, WM disruption in cervical SCI was worse than in thoracic injury level, especially in the PTR region. These results suggest that DTI imaging could be useful for elucidating mechanisms of neurodegeneration following SCI, and WM disruption may depend on injury level: the higher the injury level, the more cerebral WM integrity may suffer.

## Author Contributions

JL, YG and FG conceived the study and designed the experiments. MY, LD and DY helped in designing the experiments for better performance. YG and FG performed the experiments and wrote the manuscript. WY and ZC performed MRI scanning. YG, FG, YL and HG performed data preprocessing and statistical analysis. All authors have read and approved the final manuscript. We thank all the subjects who participated in the study for their time and cooperation.

## Conflict of Interest Statement

The authors declare that the research was conducted in the absence of any commercial or financial relationships that could be construed as a potential conflict of interest.

## Footnotes

^1^http://www.fmrib.ox.ac.uk/fsl

^2^http://cmrm.med.jhmi.edu
