# Supplementary material for: White Matter Microstructure Alterations in Patients With Spinal Cord Injury Assessed by Diffusion Tensor Imaging
Source: Front Hum Neurosci. 2019 Feb 12;13:11. doi: 10.3389/fnhum.2019.00011 (PMC6379286; doi:10.3389/fnhum.2019.00011)
Supplement: Supplementary file 1 [file Table_1.docx]

**Supplementary Table 1.**

The WM tract ROIs based on the ICBM-DTI-81 WM labels atlas within cerebral regions.

| **WM tracts** | **abbreviation** |
| --- | --- |
| Corpus callosum | CC |
| Superior longitudinal fasciculus | SLF |
| Anterior corona radiata | ACR |
| Superior corona radiata | SCR |
| Posterior corona radiata | PCR |
| Posterior thalamic radiation (include optic radiation) | PTR |
| Retrolenticular part of internal capsule | RIC |
| Sagittal stratum (include inferior longitidinal fasciculus and inferior fronto-occipital fasciculus) | SS |
| Cingulum (cingulate gyrus) | CCG |
| Posterior limb of internal capsule | PLIC |
| External capsule | EC |
| Anterior limb of internal capsule | ALIC |
| Tapetum | TAP |
| Superior fronto-occipital fasciculus (could be a part of anterior internal capsule) | SFOF |
| Cingulum (hippocampus) | CH |
| Inferior fronto-occipital fasciculus | IFOF |
